# Testing a One-Item Risk Measure to Predict Alameda Seven Health Behaviors in the Republic of Korea

**DOI:** 10.3390/ijerph18010300

**Published:** 2021-01-03

**Authors:** Donata Bessey

**Affiliations:** East Asia International College, Yonsei University, Mirae Campus, Wonju 26493, Korea; dbessey@yonsei.ac.kr; Tel.: +82-33-760-2276

**Keywords:** health behaviors, personality traits, cognitive ability, risk attitude

## Abstract

The college years provide an important window of opportunity for the implementation of preventive efforts, especially with respect to smoking, problematic drinking, and obesity. Targeting of individuals at high risk of adopting those health behaviors might increase the effectiveness of those efforts, yet little is known about possible criteria for targeting and their ability to predict the adoption of risky health behaviors. Human capital theory predicts that more risk-averse individuals are more likely to invest in their health capital and should therefore be less likely to engage in risky health behaviors. Building on this theoretical prediction, this study uses a simple one-item measure of risk attitude and examines its ability to predict the Alameda Seven health behaviors in a sample of college students in the Republic of Korea. Unlike previous research, it also controls for personality traits and cognitive ability. Experimental data were gathered and analyzed using regression analysis. The risk measure predicted the probability of smoking and reporting higher stress levels, with no correlations found for the other health behaviors, suggesting that replication studies using larger samples should be carried out to analyze if these findings persist.

## 1. Introduction

According to the World Health Organization’s latest “Global Burden of Disease” report, high systolic blood pressure, fasting plasma glucose, and body mass index were the three risk factors associated with the most pronounced increases in exposure and related losses in disability-adjusted life years (DALYs) between 2010 and 2019 [1]. These risk factors are preventable; therefore, the development of preventive efforts for lifestyle-related diseases has become a public health policy objective in many high-income countries including the United States, Australia, and Denmark [2,3,4]. In the next decades, the number of patients with those diseases is projected to increase further [5,6,7], putting an additional strain on healthcare systems.

In their college years, most students live in dorms, or other forms of housing, away from their parental home, and many engage in new health behaviors that shape their lifestyles [8,9] such as smoking [10], binge drinking [11], or eating behaviors that lead to weight gain [9] and contribute to obesity levels, particularly in the United States [12]. While initiation to and experimentation with smoking usually take place in adolescence, transition to regular use typically occurs during the college years [10]. Smoking cessation before age 30 dramatically decreases the risk of smoking-related mortality [13], suggesting that the college years are a particularly important window of opportunity for preventive efforts to be implemented.

When deciding on target populations for preventive efforts, one approach could be to develop simple markers to identify those at risk of adopting unhealthy behaviors (or unlikely to adopt healthy ones) and provide them with tailored preventive measures and programs [14]. Previous evidence, as summarized in two review articles [15,16], suggests that targeted preventive health check-ups in general practice might be beneficial for high-risk individuals. Instead of health check-ups, this research is informed by economic decision-making theory to derive a possible predictor of the probability of engaging in health behaviors. Human capital theory, developed by Becker [17] and Grossman [18], and extended to risky investments [19,20,21], provides an economic analysis of individual decisions to invest in health and predicts that more risk-averse individuals are more likely to invest in their health capital and should therefore be less likely to engage in risky health behaviors. This study uses a simple one-item measure of risk attitude (as suggested by Dohmen et al. [22], henceforth “D”) and analyzes its ability to explain the probability of adopting the Alameda Seven health behaviors (smoking, drinking excessively, exercising, following a healthy diet, being overweight or obese, experiencing stress, and sleeping enough), using data collected in an experiment. These seven health behaviors were first analyzed in 1965 in an epidemiological study in Alameda County, California. Empirical research shows that some, or all, of the Alameda Seven affect physical health status [23,24,25,26,27,28]. While there is a strand of literature in health economics that employs risk measures elicited in incentivized economic experiments or other proxy measures of risk, these measures are typically “expensive” both in terms of money and time required in surveys to be administered. Dohmen et al. [22] therefore propose a one-item risk attitude measure that is correlated with other risk measures, such as choices in incentivized lotteries on one hand, and predicts a number of different risky behaviors, such as choosing a risky occupation or choosing to smoke, on the other. The fact that D has been found to predict more risky health behaviors than other measures [29] and that its ability to predict risky health behaviors has been confirmed [30], provided the rationale to analyze its usefulness as a predictor of a full set of health behaviors and outcomes in an experiment using college students as subjects. Unlike this previous research using D to predict only a small number of risky health behaviors, this study analyzes the full set of Alameda Seven health behaviors.

Additionally, this research is related to two strands of the literature in health psychology. The first one analyzes correlations between Big Five personality traits and health behaviors [31,32,33,34,35,36,37] and the second analyzes correlations between cognitive ability and health behaviors [38,39,40,41,42]. Those strands of literature showed important effects of both ability and personality, suggesting that failure to include information on those traits could lead to omitted variable bias in regressions of the determinants of health behaviors and provide the rationale to include a measure of cognitive ability as well as information on respondents’ Big Five personality traits in regression analyses. Previous research in economics also shows a relationship between cognitive ability and risk-taking [43], adding further evidence for the need to include a measure of cognitive ability.

This study aims to test the suitability of a one-item risk measure (D) as a predictor of the probabilities of engaging in Alameda Seven health behaviors, using an experimental study approach and a sample of college students. Unlike the scant previous evidence [29,30], it also includes information on subjects’ Big Five personality traits derived from a 15-item inventory [44,45] and an ultra-short measure of cognitive ability [46,47] in regression analyses.

## 2. Materials and Methods

### 2.1. Theoretical Considerations

Human capital theory was first developed by Becker [17] with an application to education and Grossman [18] with an application to health investment, and later extended to the analysis of risky investments [19,20,21]. It provides a framework for analysis of the decision to engage in health behaviors with uncertain outcomes as risky investments in human capital. Individual risk attitudes measured using a variety of elicitation methods are a key determinant of human capital investment decisions. Human capital theory predicts that more risk-averse individuals are more likely to invest in their health capital and should therefore be less likely to engage in risky health behaviors, and are more likely to engage in beneficial health behaviors.

### 2.2. Data Collection, Experimental Methods and Questionnaires

Data were collected at a college campus in Gangwon-do, Republic of Korea. Freshmen students were recruited as subjects, with no restrictions on major, age, or gender. 

**Figure 1:**
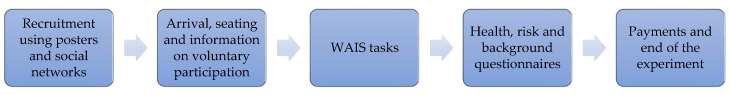
Experimental design.

After a pretest, three experimental sessions were conducted between March and April 2018, with a total of 178 subjects participating. After deleting observations with missing values, *n* = 131 subjects remained. For basic demographics of the sample and a correlation matrix, please refer to Table A2 and Table A3 in Appendix A. The following flowchart presents an overview of the experimental design (Figure 1).

In all experimental sessions, subjects first were welcomed to the experiment, and were informed about the study, the procedures, and their rights regarding voluntary participation. Next, they completed the Animal Naming Task and the Symbol Correspondence Task, which are from the Wechsler Adult Intelligence Scale (WAIS). These tasks were intended to serve as a measure of cognitive ability. Previous studies suggest that scores for these tasks are highly correlated with scores on other submodules of the WAIS and other widely used intelligence tests [46,47]; therefore, they can serve as a measure of cognitive ability without taking a long time to complete. In this research, only the scores for the Animal Naming Task could be used as a measure of cognitive ability because the majority of subjects approached the Symbol Correspondence Task in an incorrect way, i.e., instead of filling in symbols in a row, as the Symbol Correspondence Task requires, subjects first filled in one symbol for the entire task and then the next, causing the scores to be disregarded.

In the next stage of each experimental session, subjects answered a health questionnaire containing items related to the Alameda Seven health behaviors. The health behaviors included in this research were the original Alameda Seven: smoking, drinking excessively, being overweight or obese, experiencing stress, following a healthy diet, exercising, and sleeping enough. These behaviors were measured based on the subjects’ answers to questions from the Canadian Community Health Survey 2016 [48], which can be found in Appendix B.

After the health survey, subjects answered a 15-item short version of the Big Five inventory that was developed and validated for use in the German Socio-Economic Panel [44,45]. The Big Five are five dimensions which define human personality at the broadest level, based on the following descriptors of language [49,50]: extraversion, agreeableness, conscientiousness, emotional stability, and openness to experience. Previous research suggests that the Big Five personality traits predict health-related behaviors and outcomes [31,32,33,34,35,36,37].

The Big Five personality inventory was scored identically to the original research [44,45] and was not standardized. In this part of each experimental session, subjects also answered the following question: “How willing are you to take risks, in general?” [22]. Respondents rated their willingness on a scale from 1 to 10, where higher values corresponded to higher willingness to take risks. Previous studies [24,25] suggest that this risk measure predicts several risky health behaviors.

Lastly, the subjects answered a questionnaire containing items about their gender, age, and family background, and were paid for their participation in the experiment.

More detail about the experimental procedures, the tasks from the WAIS subscales, the 15-item Big Five inventory, and the health and background questionnaires are shown in Appendix B.

### 2.3. Statistical Methods

The empirical analysis of the experimental data was carried out using STATA 16.1. First, the following seven regression models were estimated: for the determinants of smoking status, a simple probit model was estimated; for the determinants of number of alcohol binges during the week prior to the experiment, level of stress, sleep quality, BMI, general health, and general mental health, ordered probit models were estimated; for the determinants of nutritional quality and minutes of exercise, ordinary least squares models were estimated. All estimations included the following control variables: gender, age, an income measure (as the answer to the question “How difficult is it for you to raise 100,000 Won for personal consumption?”, with 1 = very easy and 5 = very hard), the respondents’ number of siblings, and a measure of family background (as the answer to the question “Did you have a happy childhood?” with 1 = very unhappy and 5 = very happy). Second, predicted probabilities of engaging in the Alameda Seven health behaviors were plotted for one-step increases in the D measure. As these plots tend to become confusing for ordered outcomes (such as most of the dependent variables analyzed here), all outcomes were binarized and plots for those probit regressions are presented, at the cost of loss of information about the dependent variables. The outcomes were binarized as follows: a value of 1 was assigned for having had any binge drinking episode during the week before the experiment, for experiencing “fairly high” or “very high” stress levels, for reporting above-average sleep quality, for having a BMI above 23 (following the Korean Society for the Study of Obesity’s 2018 suggestion to create a category of “pre-obesity” for Korean individuals with a BMI above 23 and under 25 [51]), for exercising for an average of at least 30 min per day, and for having fruits or vegetables at least once daily. Predicted probabilities and the corresponding plots were calculated and graphed using the margins command in STATA 16.1, for increases in one of the D risk measures. All other variables were held constant at the sample means, with the exception of the ordered variables, which were held constant at the sample modes.

## 3. Results

Table 1 presents descriptive statistics. The average age of the subjects was 19.3 years and 76 were female. A binge drinking episode in the week before the experiment was reported by 71.76% of the participants and 11.45% reported that they were smokers. On average, the subjects reported exercising for 166 min in the week before the experiment and mean self-reported BMI in the sample was 22.1. A correlation matrix is provided in Appendix A, Table A1.

Table 2 presents results from seven regressions for the determinants of the Alameda Seven health behaviors, using the D risk measure and a full set of control variables. The estimated coefficients of interests are reported here and full regression results including all control variables can be found in the Appendix A, Table A2. In addition, results from regressions without any control variables and with only a basic set of control variables are provided in the Appendix A, Table A3 and Table A4.

The results show that D performed poorly as a predictor of Alameda Seven health behaviors and health outcomes. D was positively correlated with the probability of being a smoker, and negatively correlated with perceived stress levels.

For the control variables, cognitive ability was found to be positively correlated with nutrition quality in regressions. From the Big Five, conscientiousness was negatively correlated with BMI, extraversion was positively correlated with the probability of being a smoker, binge drinking, sleep quality, nutrition quality, minutes exercised, and general physical and mental health. Agreeableness was negatively correlated with stress levels, while neuroticism was negatively correlated with sleep quality, positively correlated with stress levels, and negatively correlated with general physical and mental health. With respect to gender differences, female students were less likely to smoke, but were more likely to report a binge drinking episode in the week prior to the experiment, had lower BMI, reported worse nutrition quality, fewer minutes exercised, and lower levels of general mental health. Age was found to be negatively correlated with BMI. Lastly, reporting a happier childhood was negatively correlated with stress, positively correlated with sleep quality, negatively correlated with BMI and minutes exercised, and positively correlated with general mental health.

**Figure 2:**
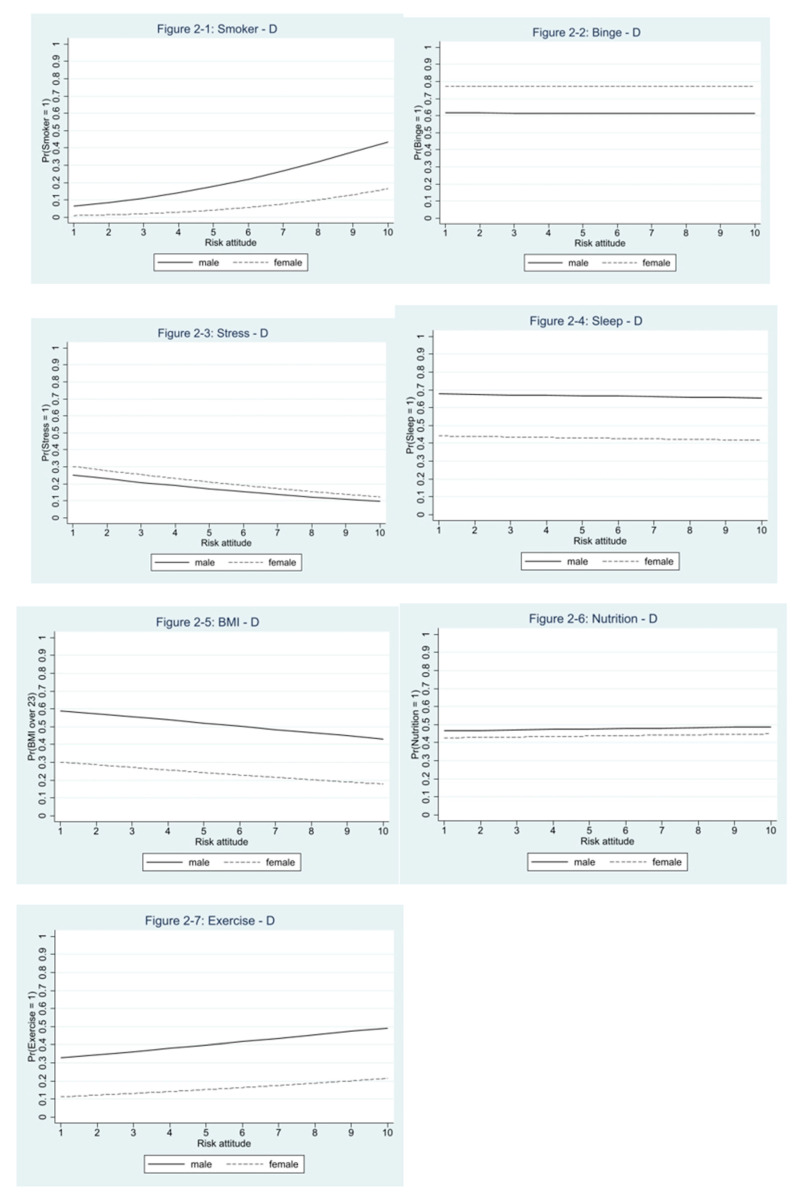
Plots of predicted probabilities by levels of D, for all Alameda Seven health behaviors.

In order to shed more light on possible nonlinearities in the relationship of D with Alameda Seven health outcomes, plots of predicted probabilities of binarized outcomes over the range of the D risk measure are presented in Figure 2. As the regression results indicate substantial gender differences, plots for male and female students are presented. 

The plots reveal that the probability of being a smoker increases with higher levels of D, for both female and male subjects. However, the probability of reporting better nutrition and more exercise also increases with higher levels of D. The probability of reporting a binge drinking episode and better quality of sleep are almost identical across the whole range of possible levels of D. Lastly, the probability of reporting high stress levels and having a BMI >23 decreases with higher levels of D. There are substantial gender differences in predicted probabilities for all Alameda Seven health behaviors.

## 4. Discussion

The aim of this study was to test the suitability of a simple one-item risk attitude measure (D) as a predictor of the health behaviors known as the Alameda Seven. When controlling for the Big Five personality traits and cognitive ability, D predicted the probability of smoking and reporting higher stress levels, with no correlations found for the other Alameda Seven health behaviors. In addition, it also found relationships between Alameda Seven health behaviors and all Big Five personality traits except openness. Szrek et al. [29] first analyzed the predictive power of D in a sample of South African health center clients and found that it predicted smoking, problematic drinking, seat belt non-use, and risky sexual behaviors. Brailovskaia et al. [30] used a German sample and found that D predicted smoking and problematic drinking. The finding that D predicts smoking was confirmed in this research, but no effects were found for drinking and the other Alameda Seven health behaviors, suggesting that it is less suitable as a predictor of health behaviors for targeted prevention programs for college students. Plots of predicted probabilities of engaging in the health behaviors analyzed in this research by levels of D revealed that the probability of smoking increases with higher levels of D, for both female and male subjects, as predicted by human capital theory. The probability of reporting a BMI >23 decreases with higher levels of D, which is also in line with the predictions of human capital theory.

However, the probability of reporting better nutrition and more exercise also increases with higher levels of D, and the probability of reporting high stress levels decreases with higher levels of D. Lastly, the probability of reporting a binge drinking episode and better quality of sleep are almost identical across the whole range of possible levels of D. These findings are not in line with the predictions of human capital theory.

With respect to the Big Five personality traits, earlier studies suggested that correlations seemed to be strongest for conscientiousness and neuroticism [34,36], but Weston et al. [37] also found relationships between extraversion, agreeableness, conscientiousness, and neuroticism with individual diseases, such as hypertension or diabetes. This research found relationships between Alameda Seven health behaviors and all Big Five personality traits except openness, confirming those previous research findings.

For cognitive ability and its possible impact on health behaviors and outcomes, Deary [52] suggested that one possible transmission pathway between early-life ability and later-life health outcome was a different probability of adopting health-related behaviors, but that “a clear chain of causation from intelligence to health outcomes and then to death has not emerged.” Auld and Sidhu [38] found that cognitive ability was positively correlated with health status; Harris et al. [41] found that childhood IQ was positively correlated with old-age health status; and Bijwaard et al. [39] found that a selection effect based on measures of ability accounted for about half of the raw differences in mortality in a Dutch sample. Wraw et al. [40] found that cognitive ability was positively correlated with physical health and negatively correlated with the probability of suffering from chronic health conditions, e.g., diabetes, between the ages of 49–55. Fawns-Ritchie et al. [42] found that cognitive ability was negatively correlated with the probability of currently smoking, but not with the probability of ever having smoked. This research found that cognitive ability was positively correlated with nutrition quality, but no relationships were found with the other Alameda Seven health behaviors or health outcomes. A tentative reason for this might be the fact that unlike in previous literature, only a one-item measure of cognitive ability was used in this study.

The previous evidence that D predicts smoking was confirmed in this research, but no relationship was found between problematic drinking and the other Alameda Seven health behaviors. The first possible explanation for this is the fact that this research used a younger sample, compared to the existing research, and as longitudinal neuroimaging studies have shown, the human brain continues to mature until about age 25 [53,54,55,56,57], leading to differences in risk perception [58,59]. This might provide a possible reason for the lack of statistically significant correlations found in this research. Finally, intercultural differences in social norms regarding risk perception [60] might provide a tentative explanation, as the previous research used samples from South Africa and Germany.

Limitations of this study include the relatively small sample size and resulting lack of statistical power, which could also provide an explanation for small number of statistically significant correlations found in this research. In addition, while college students are an important group for the targeting of preventive efforts, replication studies drawing samples from the general population should be carried out in the future to investigate if the results found in this research can be generalized. Lastly, the measure of cognitive ability used in this research consists only of one item and while this item has been shown to be highly correlated with scores on other submodules of the WAIS and other widely-used intelligence tests [46,47], more precise measures should be used in future research on the topic.

## 5. Conclusions

This research found that, when controlling for Big Five traits and including a measure of cognitive ability, D was a statistically significant predictor of only two Alameda Seven health behaviors: the probabilities of being a smoker and experiencing higher stress levels, where the correlation was positive for smoking and negative for stress.

As D is a “cheap” risk measure, both in terms of the time required for its completion in surveys and the lack of a need for financial incentives, future research could involve more testing of its predictive power of health behaviors in large-scale surveys using more diverse samples. Lastly, as Bran and Vaidis [61] have pointed out, the development of new measures of risk-taking might be a promising avenue for further research and may eventually lead to the discovery of suitable simple markers for the targeting of preventive efforts, both in college students and other populations.

## Figures and Tables

**Table 1:** Basic descriptive statistics.

	Mean	Std. Dev.	Min	Max
Last week binge (0 = never, 3 = daily)	1.1527	0.8899	0	3
Smoker (1 = yes)	0.1145	0.3196	0	1
Stress (1 = not at all, 5 = extremely stressful)	2.9008	0.7830	1	4
Sleep quality (index, 5 = high)	3.5038	0.5583	1.3333	5
Nutrition quality (times fruit/vegetables eaten)	6.4351	3.4061	0	14
Exercise (minutes)	172.5954	185.1084	0	960
BMI (weight/height²)	0.2214	0.4850	0	2
Self-rated risk tolerance D (1 = low, 10 = high)	5.2977	2.3557	1	10
Cognitive ability measure	22.9618	4.9418	12	40
Big 5 Openness	4.7939	1.1741	1.6667	7
Big 5 Conscientiousness	4.1298	0.9586	2	7
Big 5 Extraversion	4.7150	1.2363	1	7
Big 5 Agreeableness	4.8372	0.9468	2.6667	6.6667
Big 5 Neuroticism	4.6005	1.0785	1	7
Income measure (1 = very easy, 5 = very hard)	3.3817	0.9237	1	5
Female (1 = female)	0.5802	0.4954	0	1
Age (years)	19.2901	0.8273	18	24
Number of siblings	1.1603	0.6179	0	4
Happy childhood (1 = very unhappy, 5 = very happy)	3.9542	0.8025	2	5

**Table 2:** D with personality and ability measures (estimated coefficients from probit, ordered probit, and ordinary least squares regressions).

	Smoker	Binges	Stress	Sleep	BMI	Nutrition	Physical Activity
	(probit)	(oprobit)	(oprobit)	(oprobit)	(oprobit)	(OLS)	(OLS)
D	0.1641 *	0.0025	−0.1001 **	0.0051	0.0306	0.1012	0.6814
	(0.0854)	(0.0481)	(0.0502)	(0.0456)	(0.0696)	(0.1431)	(7.7992)

Notes: Standard errors are given in brackets. ** denotes 5%, and * denotes 10% significance levels.

## Data Availability

The data presented in this study are available on request from the corresponding author. The data are not publicly available due to data and privacy protection requirements.

## Appendix A

**Table A1:** Correlation matrix.

	Binge	Smoker	Stress	Sleep	Nutrition	Exercise	BMI	D	Cogn. abil.	Openn.	Consc.	Extrav.	Agree.	Neurot.	Income	Female	Age	Siblings	Childhood
Binge	1																		
Smoker	0.1270	1																	
	(0.1470)																		
Stress	0.0329	0.0150	1																
	(0.7090)	(0.8650)																	
Sleep	−0.0631	−0.1390	−0.3129 *	1															
	(0.4740)	(0.1130)	(0.0003)																
Nutrition	0.1630	0.1941 *	−0.1510	−0.0339	1														
	(0.0626)	(0.0263)	(0.0852)	(0.7010)															
Exercise	0.0394	0.0885	−0.2203 *	0.1520	0.1978 *	1													
	(0.6550)	(0.3150)	(0.0115)	(0.0836)	(0.0235)														
BMI	−0.2393*	−0.1150	0.1190	0.0489	−0.0588	−0.0292	1												
	(0.0059)	(0.1900)	(0.1760)	(0.5790)	(0.5050)	(0.7410)													
D	0.0222	0.1380	−0.3050 *	0.1330	0.0729	0.0573	0.0698	1											
	(0.8010)	(0.1150)	(0.0004)	(0.1310)	(0.4080)	(0.5160)	(0.4280)												
Cogn. abil.	−0.0022	0.0174	−0.1460	−0.0088	0.2903 *	0.0987	0.0870	0.0710	1										
	(0.9800)	(0.8440)	(0.0959)	(0.9210)	(0.0008)	(0.2620)	(0.3230)	(0.4200)											
Openn.	0.0181	0.0497	−0.0810	0.1490	0.1160	0.0640	0.0042	0.3033 *	0.2664 *	1									
	(0.8380)	(0.5730)	(0.3580)	(0.0890)	(0.1860)	(0.4680)	(0.9620)	(0.0004)	(0.0021)										
Consc.	0.0968	0.0432	−0.2252 *	0.2697 *	0.1620	0.2279 *	−0.1560	0.2065 *	0.0676	0.1330	1								
	(0.2710)	(0.6240)	(0.0097)	(0.0018)	(0.0638)	(0.0088)	(0.0752)	(0.0180)	(0.4430)	(0.1310)									
Extrav.	0.1727 *	0.1030	−0.1990 *	0.2034 *	0.2087 *	0.1690	−0.0607	0.1460	0.1050	0.1310	0.1735 *	1							
	(0.0485)	(0.2430)	(0.0227)	(0.0198)	(0.0168)	(0.0543)	(0.4910)	(0.0951)	(0.2320)	(0.1350)	(0.0475)								
Agree.	0.0358	0.0621	−0.2468 *	0.2243 *	0.1510	0.1550	−0.0382	0.1430	0.0272	0.2202 *	0.3936 *	−0.1772 *	1						
	(0.6850)	(0.4810)	(0.0045)	(0.0100)	(0.0852)	0.0765)	(0.6650)	(0.1040)	(0.7580)	(0.0115)	(0.0000)	(0.0429)							
Neurot.	0.0480	0.0519	0.3838 *	−0.2395 *	−0.0123	−0.0343	−0.0796	−0.4605 *	−0.1790*	−0.2066 *	−0.2661 *	−0.1040	0.2237 *	1					
	(0.5860)	(0.5560)	(0.0000)	(0.0059)	(0.8890)	(0.6970)	(0.3660)	(0.0000)	(0.0408)	(0.0179)	(0.0021)	(0.2360)	(0.0102)						
Income	0.0502	0.0072	0.2442 *	−0.0973	−0.0385	−0.0715	0.0160	−0.2223 *	−0.0541	−0.2059 *	−0.2070 *	−0.1852 *	0.0484	0.0839	1				
	(0.5690)	(0.9350)	(0.0049)	(0.2690)	(0.6620)	0.4170)	(0.8560)	(0.0107)	(0.5400)	(0.0183)	(0.0177)	(0.0342)	(0.5830)	(0.3410)					
Female	0.1460	−0.1798 *	0.0504	−0.0822	−0.1050	−0.2497 *	−0.1540	−0.0371	0.0688	−0.0044	−0.1975 *	0.0771	−0.1340	−0.1863 *	−0.0759	1			
	(0.0950)	(0.0398)	(0.5680)	(0.3500)	(0.2320)	0.0040)	(0.0782)	(0.6740)	(0.4350)	(0.9600)	(0.0237)	(0.3810)	(0.1280)	(0.0332)	(0.3890)				
Age	0.0543	0.0189	−0.0621	−0.0857	0.0914	−0.0306	−0.1040	0.1610	0.0498	0.1020	0.0944	0.0406	−0.0571	−0.1754 *	−0.0297	0.1942 *	1		
	(0.5380)	(0.8310)	(0.4810)	(0.3310)	(0.2990)	0.7290)	(0.2380)	(0.0669)	(0.5720)	(0.2480)	(0.2830)	(0.6450)	(0.5170)	(0.0451)	(0.7360)	(0.0262)			
Siblings	−0.0588	−0.0547	−0.0941	−0.0650	0.0653	0.0646	−0.0680	−0.0330	0.0549	0.0459	0.0166	0.2601 *	−0.0746	−0.0696	0.1250	−0.1420	−0.0782	1	
	(0.5040)	(0.5350)	(0.2850)	(0.4610)	(0.4590)	(0.4640)	(0.4400)	(0.7080)	(0.5330)	(0.6030)	(0.8510)	(0.0027)	(0.3970)	(0.4290)	(0.1540)	(0.1060)	(0.3750)		
Childhood	−0.0548	0.0506	−0.2154 *	0.2522 *	0.1060	−0.0908	−0.2109 *	−0.0334	−0.0295	0.0498	0.2144 *	0.0177	−0.1510	−0.2026*	0.2511 *	0.0714	0.0468	0.1680	1
	(0.5340)	(0.5660)	(0.0135)	(0.0037)	(0.2290)	(0.3020)	(0.0156)	(0.7050)	(0.7380)	(0.5720)	(0.0139)	(0.8410)	(0.0842)	(0.0203)	(0.0038)	(0.4180)	(0.5950)	(0.0556)	

Pairwise correlations, *p*-values are reported in parentheses, * denotes a significance level of 5%.

**Table A2:** All estimated coefficients.

	Smoker	Binges	Stress	Sleep	BMI	Nutrition	Physical Activity
	(probit)	(oprobit)	(oprobit)	(oprobit)	(oprobit)	(OLS)	(OLS)
D	0.1641 *	0.0025	−0.1001 **	0.0051	0.0306	0.1012	0.6814
	(0.0854)	(0.0481)	(0.0502)	(0.0456)	(0.0696)	(0.1431)	(7.7992)
Cognitive ability	0.0121	−0.0050	−0.0305	−0.0122	0.0231	0.2051 ***	3.3423
measure	(0.0325)	(0.0206)	(0.0213)	(0.0193)	(0.0280)	(0.0605)	(3.2998)
Big Five	0.0163	0.0149	0.1447	0.0717	0.0034	−0.0229	−0.7798
Openness	(0.1496)	(0.0924)	(0.0956)	(0.0860)	(0.1247)	(0.2692)	(14.6737)
Big Five	−0.0025	0.1717	0.0035	0.1274	−0.2898 *	0.1798	29.3221
Conscientiousness	(0.1890)	(0.1177)	(0.1203)	(0.1102)	(0.1548)	(0.3444)	(18.7706)
Big Five	0.2660 *	0.1470 *	−0.1040	0.1760 **	0.0592	0.5832 **	35.8987 **
Extraversion	(0.1587)	(0.0858)	(0.0875)	(0.0810)	(0.1144)	(0.2516)	(13.7120)
Big Five	0.0017	0.0750	−0.1998 *	0.1223	0.0888	0.2833	20.0640
Agreeableness	(0.1868)	(0.1170)	(0.1199)	(0.1099)	(0.1725)	(0.3440)	(18.7537)
Big Five	0.2874	0.1055	0.2754 **	−0.1721 *	−0.1194	0.4543	11.2191
Neuroticism	(0.1761)	(0.1065)	(0.1101)	(0.0990)	(0.1454)	(0.3085)	(16.8147)
Income measure	0.1876	0.0868	0.1668	0.0270	0.0032	0.2291	1.5024
	(0.1959)	(0.1162)	(0.1183)	(0.1068)	(0.1545)	(0.3352)	(18.2700)
1 = female	−0.8806 **	0.3595 *	0.1272	−0.2525	−0.5666 *	−1.1696 *	−96.2704 ***
	(0.3507)	(0.2141)	(0.2186)	(0.2013)	(0.2901)	(0.6269)	(34.1706)
Age	0.0177	0.0772	0.0237	−0.1520	−0.3366 *	0.2592	−26.2763
	(0.2147)	(0.1211)	(0.1269)	(0.1163)	(0.2016)	(0.3619)	(19.7291)
Number of	−0.0785	−0.1315	−0.1289	−0.0888	−0.1888	0.2606	14.9250
siblings	(0.2997)	(0.1589)	(0.1671)	(0.1520)	(0.2420)	(0.4764)	(25.9677)
Childhood	0.2435	−0.1847	−0.2707 **	0.2238 *	−0.3457 **	0.4226	−37.4131 *
happiness	(0.2334)	(0.1327)	(0.1372)	(0.1247)	(0.1746)	(0.3885)	(21.1765)
/cut1		2.7226	−3.3304	−3.7321	−7.7240 *		
		(2.7144)	(2.8490)	(2.6212)	(4.1584)		
/cut2		3.6324	−1.4162	−3.3807	−6.5758		
		(2.7195)	(2.8035)	(2.5921)	(4.1450)		
/cut3		5.0991 *	0.0421	−2.4406			
		(2.7335)	(2.8086)	(2.5663)			
/cut4				−1.7217			
				(2.5614)			
/cut5				−1.0213			
				(2.5588)			
/cut6				−0.1574			
				(2.5570)			
/cut7				0.6064			
				(2.5595)			
/cut8				0.8429			
				(2.5633)			
/cut9				1.7038			
				(2.6092)			
Constant	−6.5494					−12.7238	345.2195
	(4.9922)					(7.9941)	(435.7548)
Observations	131	131	131	131	131	131	131

***, **, and * denote significance levels of 1%, 5%, and 10%, respectively.

**Table A3:** Regressions with only basic background control variables.

	Smoker	Binges	Stress	Sleep	BMI	Nutrition	Physical Activity
	(probit)	(oprobit)	(oprobit)	(oprobit)	(oprobit)	(OLS)	(OLS)
D	0.1178 *	0.0079	−0.1455 ***	0.0692 *	0.0420	0.0968	4.0141
	(0.0713)	(0.0417)	(0.0439)	(0.0398)	(0.0597)	(0.1321)	(7.0377)
Income measure	0.1220	0.0359	0.1910 *	−0.0565	−0.0266	0.0824	−10.1302
	(0.1722)	(0.1110)	(0.1130)	(0.1034)	(0.1453)	(0.3442)	(18.3440)
1 = female	−0.7253 **	0.3481 *	0.1285	−0.2420	−0.4405 *	−0.7835	−89.5387 ***
	(0.3212)	(0.1963)	(0.1989)	(0.1850)	(0.2656)	(0.6121)	(32.6207)
Age	0.0003	0.0954	−0.0117	−0.0982	−0.3378 *	0.3455	−19.7821
	(0.1966)	(0.1198)	(0.1242)	(0.1149)	(0.1953)	(0.3811)	(20.3105)
Number of siblings	−0.1499	−0.1223	−0.1739	−0.0505	−0.1702	0.3458	18.5647
	(0.2905)	(0.1578)	(0.1635)	(0.1509)	(0.2307)	(0.5027)	(26.7876)
Childhood happiness	0.2122	−0.0899	−0.3500 ***	0.3516 ***	−0.3826 **	0.5974	−16.2212
	(0.2055)	(0.1224)	(0.1269)	(0.1170)	(0.1614)	(0.3827)	(20.3930)
/cut1		1.1213	−4.1533	−3.1075	−7.3721 *		
		(2.4855)	(2.6236)	(2.4329)	(4.0314)		
/cut2		2.0000	−2.4039	−2.8038	−6.2886		
		(2.4890)	(2.5828)	(2.4103)	(4.0232)		
/cut3		3.4238	−1.0481	−1.9376			
		(2.4983)	(2.5834)	(2.3896)			
/cut4				−1.2472			
				(2.3856)			
/cut5				−0.5806			
				(2.3833)			
/cut6				0.2176			
				(2.3810)			
/cut7				0.9349			
				(2.3842)			
/cut8				1.1719			
				(2.3888)			
/cut9				2.0318			
				(2.4373)			
Constant	−2.6225					−3.3303	661.7326
	(4.0147)					(7.9052)	(421.2722)
Observations	131	131	131	131	131	131	131

***, **, and * denote significance levels of 1%, 5%, and 10%, respectively.

**Table A4:** Regressions with only D.

	Smoker	Binges	Stress	Sleep	BMI	Nutrition	Physical Activity
	(probit)	(oprobit)	(oprobit)	(oprobit)	(oprobit)	(OLS)	(OLS)
D	0.1021	0.0095	−0.1508 ***	0.0635 *	0.0463	0.1054	4.5035
	(0.0644)	(0.0401)	(0.0421)	(0.0382)	(0.0539)	(0.1270)	(6.9072)
/cut1		−0.5258 **	−2.9365 ***	−2.1037 ***	1.1257 ***		
		(0.2412)	(0.3728)	(0.4118)	(0.3215)		
/cut2		0.3311	−1.3114 ***	−1.8374 ***	2.1280 ***		
		(0.2397)	(0.2591)	(0.3397)	(0.3708)		
/cut3		1.7377 ***	−0.0399	−1.0084 ***			
		(0.2871)	(0.2434)	(0.2473)			
/cut4				−0.3415			
				(0.2300)			
/cut5				0.2872			
				(0.2296)			
/cut6				1.0368 ***			
				(0.2396)			
/cut7				1.7320 ***			
				(0.2653)			
/cut8				1.9670 ***			
				(0.2806)			
/cut9				2.7982 ***			
				(0.4334)			
Constant	−1.7784 ***					5.8768 ***	148.7372 ***
	(0.4031)					(0.7357)	(40.0217)
Observations	131	131	131	131	131	131	131

***, **, and * denote significance levels of 1%, 5%, and 10%, respectively.

## Appendix B. Experimental Procedures, WAIS Tasks, Big Five Personality Trait Items, Health and Background Questionnaires

All data used in this research were collected in a paper-and-pencil experiment. Subjects were freshmen students on a college campus in the Republic of Korea who were recruited using posters on campus and social network services.

In all three experimental sessions, subjects were seated apart and then welcomed to the experiment. The first two tasks were the Animal Naming Task and the Symbol Correspondence Test, both taken from the Wechsler Adult Intelligence Scale, although the scores from the Symbol Correspondence Test could not be used for analysis.

Task 1: In the following 90 s, write down as many animals as you can.

Task 2: In the following, you see symbols corresponding to numbers. Please fill in the symbols in the grid below. You have 90 s.

**Personality traits measurement**

The following section includes the wording of all questions used for Big Five personality traits measurement.

In the next section, we list several characteristics that an individual might have. Most likely, you will completely agree that some characteristics describe you well, while others don’t, and you will be undecided on still others. Again, please indicate the degree to which the following statements are true for you, on a scale from 1 (not like me at all) to 7 (very much like me).

*I am a person who...*

Does a thorough job

Is talkative

Is sometimes rude to others

Is original, comes up with new ideas

Worries a lot

Has a forgiving nature

Tends to be lazy

Is outgoing, sociable

Values aesthetic, artistic experiences

Gets nervous easily

Does things efficiently

Is reserved

Is considerate and kind to almost everyone

Has an active imagination

Is relaxed, handles stress well

**Questionnaire on health behaviors and outcomes**

*In general, would you say your health is... ?*

1: Excellent

2: Very good

3: Good

4: Fair

5: Poor

*In general, would you say your mental health is...?*

1: Excellent

2: Very good

3: Good

4: Fair

5: Poor

**Stress**

*Thinking about the amount of stress in your life, would you say that most of your days are...?*

1: Not at all stressful

2: Not very stressful

3: A bit stressful

4: Quite a bit stressful

5: Extremely stressful

**Height/Weight**

*How tall are you without shoes on?*

*How much do you weigh without clothes on?*

**Sleep**

*How often do you have trouble going to sleep or staying asleep?*

1: Never

2: Rarely

3: Sometimes

4: Most of the time

5: All of the time

*How often do you find your sleep refreshing?*

1: Never

2: Rarely

3: Sometimes

4: Most of the time

5: All of the time

*How often do you find it difficult to stay awake when you want to?*

1: Never

2: Rarely

3: Sometimes

4: Most of the time

5: All of the time

**Smoking/nicotine consumption**

*At the present time, do you smoke cigarettes (including e-cigarettes) every day, occasionally or not at all?*

1: Daily

2: Occasionally

3: Not at all

**Alcohol**

*Now, some questions about your alcohol consumption.*

A ‘drink’ refers to:

- a bottle or small can of beer, cider or cooler with 5% alcohol content, or a small draft;

- a glass of wine with 12% alcohol content;

- a glass or cocktail containing 1 oz. of a spirit with 40% alcohol content.

*How often in the last week have you had [5 for males/4 for females] or more drinks on one occasion?*

1: Never

2: Once

3: More than once

4: Every day

**Physical activity**

*In the last 7 days, on which days did you do these recreational activities that made you sweat at least a little and breathe harder? Please only include activities that lasted a minimum of 10 continuous minutes.*

1: Monday: minutes

2: Tuesday: minutes

3: Wednesday: minutes

4: Thursday: minutes

5: Friday: minutes

6: Saturday: minutes

7: Sunday: minutes

**Nutrition**

*During the last week, on how many days have you eaten vegetables or have you drunk vegetable juice?*

*During the last week, on how many days have you eaten fruit or have you drunk fruit juice?*

For all questions:

never

one day

two days

three days

four days

five days

six days

every day

**Background questionnaire**

Please answer the following questions about yourself.

*How difficult would it be for you to raise 100,000 Won for personal consumption?*

Very difficult

Difficult

Neutral

Easy

Very easy

*What is your major?*

*What is your gender? Female  □  Male  □  Prefer not to say  □*

*What is your year of birth?*

*Do you have siblings? If yes, how many?*

*On a scale from 1 to 5, how happy would you say your childhood was?*

Very unhappy

Unhappy

Neither happy nor unhappy

Happy

Very happy

*How willing are you to take risks, in general? Please rate your willingness to take risks on a scale from 1 to 10, where 1 corresponds to low and 10 corresponds to high willingness to take risks.*
